# Parental Approach to the Management of Childhood Fever: Differences between Health Professional and Non-Health Professional Parents

**DOI:** 10.3390/ijerph16204014

**Published:** 2019-10-20

**Authors:** María Gloria Villarejo-Rodríguez, Beatriz Rodríguez-Martín

**Affiliations:** 1 Health Center in Bargas, 45593 Toledo, Spain; 2 Faculty of Health Sciences, University of Castilla-La Mancha, Talavera de la Reina, 45600 Toledo, Spain; Beatriz.RMartin@uclm.es

**Keywords:** child, fever, qualitative research, management, parents

## Abstract

Fever is responsible for 30% of pediatric consultations at primary care services. The aim of this study was to explore the parental approach to fever in children aged between 0 and 12 years old by both health professional and non-health professional parents. A qualitative study based on grounded theory was performed. Focus groups were conducted, segmented by sex, place of residence, and healthcare training, using a triangulated sample (theoretical and snowball sampling) of parents of children aged between 0 and 12 years who were treated for fever at primary care emergency services. The constant comparative method and a process of coding was used for the analysis. The study findings reveal that the health training of parents, their former experience, family pressures, the age of the child, and the parents’ work outside the home, all influenced how they approached fever management. These findings could be incorporated into clinical practice to improve care and compliance with fever treatment.

## 1. Introduction

Fever, defined as a rectal temperature above 38 degrees centigrade (°C) [1], is the body’s response to an illness or pathological invasion [2] and is one of the most common symptoms of childhood illnesses (for one-third of all presenting conditions in children) [3]. Generally, fever is self-limiting and a symptom of a banal viral infection with no known source [4].

Despite this, childhood fever constitutes 30% of pediatric consultations in primary care in both Europe and the United States [5,6]. In Spain, reported rates are as high as 60% [7]. This represents a considerable expense considering the extent of medical consultations and emergency service visits in primary care sectors and hospitals, together with complementary exams and antibiotic treatments, which, at times, could be deemed unnecessary.

Regarding the manner in which parents approach childhood fever, prior studies show that parents fear the secondary effects of fever, such as febrile convulsions or brain injury [8,9,10], or the association between high fever and the severity of the illness [11,12]. These are factors which are conducive to parents initiating antipyretic therapy as soon as possible, thus increasing the risk of an overdose [13]. In this sense, according to previous studies, approximately 50% of parents administer antipyretics when temperatures are below 38 °C or administer incorrect doses of antipyretics or supratherapeutic doses of paracetamol and ibuprofen [14,15]. It is therefore worth considering that parent empowerment in the management of fever could be a helpful tool for gaining greater control over the decisions and actions that affect both parents’ health and that of their children. Parent empowerment also enables parents to actively take charge of their children’s care [16,17].

Previous studies have highlighted the fact that the educational level or, more specifically, health alphabetization, influences the perceptions related to the process of health-illness in the community [18]. Moreover, parents who are health professionals can encounter difficulties when assuming the role of the patient, frequently resorting to self-diagnosis, self-medication, and prescriptions issued to family members [19,20,21]. Despite this, to the best of our knowledge, the influence of health training on parents, regarding the management of childhood fever, has not been analyzed to date.

Although the management of childhood fever is well known based on former quantitative research performed using surveys or questionnaires, little is known of the parents’ perception on the process of seeking care, the therapeutic approach, and the expectations regarding this phenomenon. These aspects can provide health professionals and managers with key information for the design and implementation of educational programs targeted at improving the manner in which parents deal with their episodes of childhood fever and promoting an appropriate use of health resources.

The aim of this study was to explore the parents’ approach to fever in children aged between 0 and 12 years, comparing parents with and without health training.

## 2. Methods 

This qualitative study based on Grounded Theory used inductive analysis, meaning that this approach primarily involved an in-depth reading of the raw data to systematically derive concepts and themes, or to construct a model based on interpretations made from the raw data on behalf of an evaluator or researcher. In Grounded Theory, the constant comparative method and coding are processes used for data analysis [22].

### 2.1. Participants/Sample

We used a triangulated sample. First, theoretical sampling was performed, guided by the constant comparison method [23,24]. The parents who participated were of Spanish nationality, with children aged between 0 and 12 years, who attended the primary care emergency service of the health centers Bargas and Buenavista; rural and urban areas of the province of Toledo for fever consultations between 1 November 2016 and 31 October 2017. We included parents both with and without health training. This study excluded parents of children with a background in respiratory disorders, such as asthma, pneumonia, or bronchiolitis. In addition, snowball sampling methods were used to select parents of children who were active or inactive health professionals in the public or private health system and who fulfilled the inclusion criteria. The sampling process continued until the criteria of data saturation was fulfilled, which is when to continue to increase the sample would have led to information being repeated [25].

The initial contact with participants took place via telephone over a three-week period following the emergency room consultation, and was administered by pediatric nurses at the designated health clinics to explain personal goals and reasons for conducting this study.

### 2.2. Data Collection

The chosen data collection technique was focus groups. These were conducted between January and October 2017 in a calm and private setting. Each focus group comprised a maximum of ten participants (half of whom were first time parents), with an average duration of 50 min. The focus groups were audio recorded prior to obtaining written consent by participants. The groups were conducted by a moderator who used a guide with themes that could appear during the sessions and which was refined throughout the study. An observer monitored the groups and made field notes. Both researchers had training and experience in qualitative research (M.G.V.-R. and M.J.).

For the organization of the focus groups, intragroup homogeneity and heterogeneity criteria were considered [26], segmenting the sample by parents with and without health training, area of residence (rural and urban), and gender, due to the possible influence of the same as an inhibitory element, as reported in previous studies [27]. Table 1 describes the characteristics of the participants.

### 2.3. Data Analysis

The recorded material was transcribed verbatim, line by line, with F4 software and anonymized. Thereafter, the transcriptions were returned to the participants by email to obtain validation. To evaluate the point of data saturation, each transcription was analyzed prior to conducting the next focus group.

During the analysis, two researchers independently (MGVR and BRM) identified and classified the themes and coded the content of the discourse. The process of open coding enabled the extraction of concepts to group into categories. During the axial coding, the data were related, regrouped, and categorized, linking categories into subcategories. Finally, by using selective coding a main theoretical framework was established, which integrated and organized the previously identified main categories into a central category [28], after which they discussed the results to obtain consensus.

The process of coding was supported by Atlas.ti 7.0 software (ATLAS.ti Scientific Software Development GmbH, Berlin, Germany), following a flexible, circular logic, using the constant comparison method [23].

### 2.4. Ethical Considerations

This study obtained approval by the Clinical Research Ethics Committee of Hospital Complex of Toledo (nº 29 29/02/2016). All participants signed informed consent ensuring the confidentiality and anonymity according to the Organic Law 15/1999 on Personal Data Protection, of December 13 [29]. 

## 3. Results

Data saturation was achieved after the performance of eight focus groups (57 participants) (Table 2).

**Figure 1:**
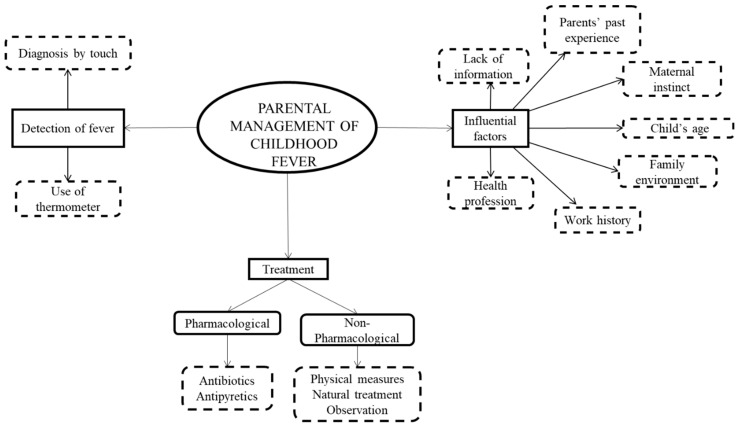
The boxes with continuous lines feature the main study themes and the boxes with dotted lines represent the categories.

The parents’ perceptions on the management of episodes of childhood fever were grouped according to three main categories: Methods for fever detection, pharmacological and non-pharmacological treatment of fever, and determinant factors for the management of febrile episodes (Figure 1). This information is presented in Table S1, in which the categories, subcategories, and emerging codes are displayed, together with the most noteworthy quotes from the focus groups and the group they belong to (non-health trained or health trained, and rural or urban area).

### 3.1. Methods for Fever Detection 

No differences were found in the methods of detection for childhood fever used by parents who resided in the urban or rural setting or according to their health training. Most parents (regardless of their health training) stated that their method of fever diagnosis was based on contact with the child’s skin, for example, via a kiss on the forehead. In addition, some participants used the thermometer to determine the exact temperature, especially when there was a suspicion of a high fever. Regarding the type of thermometer used, despite acknowledging the toxicity of mercury thermometers, parents of both groups preferred these to digital thermometers, considering that digital thermometers were unreliable. Regarding the body area used to measure fever, no parents used a rectal thermometer, rather, the temperature was measured in the armpit, on the forehead, or in the ear in the case of digital temperatures. Some non-health profession mothers also used temperature sensitive strips with color codes which were placed on the forehead to determine the presence of fever.

### 3.2. Fever Treatment

During febrile episodes, parents used pharmacological and non-pharmacological treatments for fever. 

#### 3.2.1. Pharmacological Treatment 

The parents of both groups used antibiotics with a medical prescription to resolve febrile episodes, although the health professional parents acknowledged a certain reluctance to administer the same due to their knowledge of the possible secondary effects.

In addition, the use of antipyretics was common among the parents with health training as well as those without. Most often, ibuprofen and paracetamol were used. Other less commonly used antipyretics were apirofen and metamizole. The latter was mainly used by health professionals. Health professional parents administered antipyretics only when the child had a high fever, whereas among non-health professional parents there was a tendency to also use these to treat a low-grade fever due to a fear of further fever complications (convulsions or brain damage). No differences according to the parents’ health training was also found regarding the use of antipyretics when the child was in discomfort, pain, was “fussy”, “acting silly”, irritable, or tired. On the other hand, most participants in both groups used a combined therapy with antipyretics, especially paracetamol or ibuprofen. In addition, they recognized respecting the timetable and interval of the doses (6–8 hours), and the dosage was personalized according to the child’s weight and age, although mothers from a rural setting, irrespective of their training, acknowledged that at times they administered medication using a blind guess (i.e., not using exact measurements). Furthermore, differences were observed regarding the route of administration of antipyretics. For example, for parents from rural settings, there was a tendency to administer the antipyretics rectally (in the form of suppositories). All parents highlighted that the flavor of the oral antipyretic was influential in whether the children accepted or rejected the medicine, emphasizing that children preferred the taste of ibuprofen when compared to paracetamol.

Concerning the parents’ perception of the negative effects of antipyretic medication, although both groups highlighted the possibility of medication intoxication, we found differences among the groups of parents. Thus, whereas parents with health training noted that hypothermia or allergic reactions were secondary effects of antipyretics, non-health professional parents highlighted the following: A decreased effectiveness of vaccines, the potential of antipyretics to mask the cause of fever, a weakening of the child’s defenses, or gastric damage.

#### 3.2.2. Non-Pharmacological Treatment 

Parents of both groups highlighted the following physical measures for the treatment of fever episodes: Rubs, lukewarm baths, the application of cold compresses on the forehead, groin and underarm, avoiding drafts, and changing the child’s clothes in order to lower the temperature. Besides, the parents from rural settings considered it was important to maintain an appropriate hydration during the febrile episode. Even though physical measures were common in the treatment of fever, only health professional parents considered that physical measures were the treatment of choice prior to the use of antipyretics.

Alternative or natural therapies were considered by parents without health training and from a rural setting, as being an alternative to pharmacological treatment. They acknowledged that they resorted to vitamin complexes, propolis, or other substances extracted from medicinal plants, such as belladonna. In some cases, these parents also used homeopathy or reflexology. These alternative methods were rejected by the health professional parents. 

Regarding the observation and vigilance of fever at home, the participants from urban settings, both health professionals and non-health professionals, acknowledged practicing co-sleeping (sleeping in the same bed as their children) during febrile episodes, which provided them with comfort and reassurance.

### 3.3. Determinant Factors in Parents’ Management of Fever

The primary determinant factor for the management of fever by parents was their health knowledge. The non-healthcare participants requested the need for professionals to provide them with greater information on fever, recognizing their gaps in knowledge regarding treatment or management.

All participants emphasized that self-learning, based on previous experiences, increased their perception of control when facing a febrile episode. In contrast, first-time parents felt at a disadvantage when compared with experienced parents, realizing their inexperience when it came to the management of fever in their child.

In the case of parents with previous experiences, two extreme attitudes towards fever were observed: An overprotective attitude with their children or excessive trust or “carelessness”. The latter was mainly displayed among health professional parents.

All participants, both health professionals and non-health professionals, considered that the child’s age influenced the management of fever as children of a young age (less than one year) are unable to verbalize their symptoms, plus there is a symptomatic specificity according to age, and a realization by all the participants that younger children are more vulnerable. 

In addition, participants of both groups considered that the pressure of the family environment could, both positively and negatively, influence the management of fever. In this sense, the grandparents were considered as being helpful in caring for children while, at the same time, representing a source of pressure with regards decision making and “information overload”.

Regarding the care of the child with fever, certain participants, with no differences according to their health training, believed that the father’s role had changed, considering that the care of a child with fever should be a task shared by both parents. Despite the above, some participants mentioned the existence of an innate quality in women, a so-called “maternal instinct” giving mothers a clear advantage over fathers in the management of fever.

Regarding the influence of the parents’ profession on the approach of fever, mothers who worked outside the home, both in the rural and urban contexts, considered that their work was an obstacle for caring for children with fever, as, during their workday and faced with the impossibility of the child attending a nursery/school, they must delegate the care of their children to their parents or another family member. In addition, health professional parents acknowledged diagnosing and prescribing the treatment of their own children feeling that, because of their training, they had an extra responsibility when compared to the other parents who were lacking this training.

## 4. Discussion 

To the best of our knowledge, this is the first study to compare the management of childhood fever in both health professional and non-health professional parents. The health training of parents and their knowledge on the secondary effects of drugs, influences their approach to fever and their perception, as well as their greater responsibility towards providing care by assuming both the diagnosis and the treatment of their children. Other factors which influence the approach to childhood fever are the child’s age, the previous experiences of parents, family pressures, and working outside the home.

Once fever is detected, by touch or confirmed with a thermometer, parents tend to administer antipyretics rapidly (mainly paracetamol or ibuprofen) due to a fear of complications, although recognizing the related secondary effects (weakening of the child’s defenses, decreased efficacy of vaccines, masking the cause of the fever, or causing possible allergic reactions). Health professional parents limit the use of these drugs in cases of high fever. Furthermore, aside from the fact that parents recognize the possibility of medication intoxications, some affirm that they administer medication by merely administering the approximate dose without taking accurate measurements. Another frequently requested treatment for fever, via medical prescription, are antibiotics, although health professional parents are reluctant to administer the same.

Parents commonly use physical measures to control fever, however these are only considered as the first option by health professional parents. Furthermore, non-health professional parents and those from a rural setting also used other natural and alternative treatments; these are generally shunned by health professional parents.

As noted in previous studies, parents used thermometers and skin contact as methods of fever diagnosis [30,31]. Furthermore, our results reveal that parents use different types of thermometers: Digitals, laser, reactive strips, or mercury thermometers, with the latter being preferred by parents as these are recognized as being more reliable, despite acknowledging the harmful effects of the same and the fact that these have been withdrawn from the market.

Concerning the treatment of fever, as in a previous study [32], parents mainly administered antibiotics, although our results highlight an overall reluctance for prescribing the same. Furthermore, the results of this study reveal that health professional parents only use antipyretics in cases of an established fever, whereas non-health professional parents administer the same with the slightest suspicion of fever. Paracetamol and ibuprofen were the most commonly used antipyretics, independent of the parents’ training, as in previous studies [33], whereas metamizole was mainly used by health professional parents. Furthermore, coinciding with other studies, the participants acknowledged the adverse effects of antipyretics, such as gastric damage (ibuprofen) [16,34] or drug intoxication due to excessive or incorrect use of the same [15,35]. 

In this study, we found that parents used physical measures to reduce body temperature, such as lukewarm baths or cold-water compresses, despite the scarce or insufficient evidence on the effects of body cooling for the reduction of fever [36]. This study also includes co-sleeping as a way to observe and monitor childhood fever at night, which was a common practice among both health professional and non-health professional participants. 

Previous studies show that having past experiences of a positive resolution of fever may reduce fear in parents [31] by helping them to acquire knowledge and increased strategies to resolve their child’s fever. Not being a first-time parent is also considered a determinant factor for the management of fever in children [12,18]. This study supports previous studies stating that the knowledge acquired by first-hand experience and working outside the home influence the way parents manage of fever [31,37]. In addition, we found that both the influence of family pressure and the age of the child were determinant factors regarding the approach of fever. With regard to the former, the presence of family support, such as grandparents as secondary carers, have been previously identified [12,38] as being an influential factor on the care of the child. 

Concerning the roles associated to the care of a child with fever, this study reveals that parents feel that the care of the child with fever is a task that both parents must share, which is a finding that may be related to social desirability bias. Despite this, participants consider that mothers, due to their maternal instinct, have innate qualities for caring. Likewise, for the first time, this study associates the role of mothers as having innate qualities as carers due to their maternal instinct. Furthermore, this study highlights health professional parents’ perceptions of having a greater responsibility in care due to the knowledge derived from their medical training. Another significant finding is that parents with healthcare training become responsible for the diagnosis and treatment of their children, despite this being against what is ethically recommended [20]. 

## 5. Study Strengths and Limitations 

This study followed the consolidated criteria for reporting qualitative research (COREQ) (Table S2) [39]. In addition, rigor was guaranteed via verification techniques by Morse [40]. Constant comparison and triangulation methods used by researchers and researcher triangulation and data triangulation guaranteed the validity and trustworthiness of the findings [23,24,41].

Data veracity and credibility, considered as being the main criteria for the assessment of the scientific rigor of this study, were ensured based on the literal transcription of the focus groups and the use of participant validation. 

One of the possible limitations of this study is the memory bias of participants due to the time between fever consultation for their children and the performance of the focus groups. To avoid this, the time span was limited to three months. 

## 6. Conclusions

In cases of fever, parents tend to rapidly administer antipyretics for fear of complications. Although the use of physical measures is common, only health professional parents consider these to be the first option. The use of antibiotics is also common, although health professional parents display a certain reluctance towards the administration of the same. In addition, among non-health professional parents from rural areas, there is a tendency to use natural or alternative therapies, however these are rejected by health professional parents.

The following factors influence the parents’ management of childhood fever: Having previous experience, the child’s age, having support from the family environment, responsibility shared by the parents, work of mothers outside the home, and the parents’ health training. Health professional parents perceive that they have an extra responsibility assuming the diagnosis and treatment of childhood fever.

## Figures and Tables

**Table 1:** Principal characteristics of parents participating in the focus groups.

Sex			27 Men		30 Women	
**Age**	<30 years		0		4	
	30–40 years		15		14	
	>40 years		12		12	
**Civil status**	Single		0		0	
	Married		26		30	
	Divorced		1		0	
**Number of children**	1		7		12	
	2		11		13	
	3–4		7		5	
	>4		2		0	
**Level of studies**	No studies		0		0	
	Primary studies		4		2	
	Secondary studies		6		9	
	University education: diploma, degree, bachelor’s degree		16		18	
	Master/doctorate		1		1	
**Place of residence (environment)**	Rural		13		16	
	Urban		14		14	
**Profession**			Active	Not active	Active	Not active
	Non-health professional		11	3	10	5
	Health professional	Doctor	7	0	7	0
		Nurse	4	0	8	0
		Nurse aid/Health technician	2	0	0	0

**Table 2:** Organization of the focus groups.

		Parents with Health Education	Parents Without Health Education
Rural area	Mothers	8	8
	Fathers	6	7
Urban area	Mothers	7	7
	Fathers	7	7

## Supplementary Materials

The following are available online at https://www.mdpi.com/1660-4601/16/20/4014/s1, Table S1: Themes, categories and codes, Table S2: Consolidated criteria for reporting qualitative research (COREQ).
