# Supplementary material for: Parental Approach to the Management of Childhood Fever: Differences between Health Professional and Non-Health Professional Parents
Source: Int J Environ Res Public Health. 2019 Oct 20;16(20):4014. doi: 10.3390/ijerph16204014 (PMC6844131; doi:10.3390/ijerph16204014)
Supplement: Supplementary file 1 [file ijerph-16-04014-s001.zip › table S1. Themes, categories and codes..docx]

Table 3. Theme, categories and codes for the management of fever

| THEME | CATEGORY | SUBCATEGORY | CODE | VERBALIZATIONS |
| --- | --- | --- | --- | --- |
| Detection of fever | Diagnosis by touch |  | Forehead | “When I realize that they have a slight fever, I can feel it by touching their forehead” (Mother H; GNHPM, RE) |
|  |  |  | Body | “Well, often, you don’t need to use the thermometer, just by touching them …” (Mother T; GHPM, UE) |
|  | Use of thermometer |  | Types: Mercury/ fever strip thermometer/ digital/ laser | “They were toxic [mercury thermometers]” (Mother I; GNHPM, UE) |
|  |  |  |  | “I remember that my mother had it at home… Strips where it said: No fever… it was like a black strip, and it turned red if there was a fever and if there wasn’t one it went blue. Those are great” (Mother L; GNHPM, UE) |
|  |  |  |  | “we weren’t convinced [by digital thermometers] because every time you used it a different reading came up” “but we used the digital thermometers for the underarm more than those for the forehead, ear, etc.” (Father k; GHPF, UE) |
|  |  |  | Preferences | “With the mercury thermometer it seems that you can control the temperature better compared to the new digital thermometers” (Father q, GHPF, RE) |
| Pharmacological treatment of fever | Antibiotics |  | Medical prescription | “They have been given antibiotics once, I follow the recommendations given to me by the doctor” (Father i; GHPF, UE) |
|  |  |  | Pro-Antibiotics | “They gave him an antibiotic and with the antibiotic the fever went down” (Mother B; GNHPM, RE) |
|  |  |  | Zero Antibiotics | “I am not really a fan of antibiotics… holding on” (Father p, GHPF, RE) |
|  |  |  |  | “I don’t really agree with antibiotics, zero antibiotics” (Mother α; GHPM, RE) |
|  | Antipyretics | Active ingredient | Ibuprofen | “Dalsy, that’s all you need, or Espidifen” (Mother E; GNHPM, RE) |
|  |  |  | Paracetamol | “I give him paracetamol. I begin with paracetamol.” (Mother W; GHPM, RE) |
|  |  |  | Apirofen | “Apirofen is the medicine that doesn’t make her throw-up” (Mother T; GHPM, UE) |
|  |  |  | Metamizole | “Metamizole works really well” (Mother V; GHPM, RE) |
|  |  | Indications of antipyretics | “Acting silly” | “They give it to her because, well, she is acting silly” (Mother A; GNHPM, RE) |
|  |  |  | “fussy” | “…or they are fussy [and they are given Paracetamol]” (Mother F; GNHPM, RE) |
|  |  |  | Irritable child | “If they ask for it [Paracetamol] and you ask: do they have a fever? No, it’s because today they are more irritable” (Mother B; GNHPM, RE) |
|  |  |  | Tiredness/ | “When he has a runny nose and he acts tired, I give him Paracetamol” (Mother I; GNHPM, UE) |
|  |  |  | High/sustained fever/ | “When the fever is higher than 38, otherwise, no.” “Paracetamol if it’s only fever, if he only has fever and his throat hurts, then I give him the other one to reduce swelling” (Mother ß; GHPM, RE) |
|  |  |  | Low-grade fever, not waiting | ““we don’t let it go up to 38, we immediately give him Ibuprofen” “as soon as he reaches 37...we are afraid of seizures” (Father v; GNHPF, UE) |
|  |  |  | discomfort/pain | “You give her Ibuprofen so that she can be more comfortable” “if they have a headache or something, I give them Ibuprofen or paracetamol” (Father y; GNHPF, UE) |
|  |  | Negative effects | Intoxication | “If he gets an intoxication with Paracetamol” (Mother X; GHPM, RE) |
|  |  |  | Allergic reaction | “Metamizole gave me an allergic reaction” (Mother W; GHPM, RE) |
|  |  |  | Masking the cause | “You’re masking it with the medicine” (Mother J; GNHPM, UE) |
|  |  |  | Gastric damage | “A bit of gastroenteritis or diarrhea after 2-3 days of ibuprofen” (Father z; GNHPF, UE) |
|  |  |  | Attacking the defenses | “If you get the fever down, they stop fighting it” (Mother N; GNHPM, UE) |
|  |  |  | Decreased effectiveness of vaccines | “When they give him vaccines, they tell you: if he doesn’t have a high fever don’t give it to him (the antipyretic) because then it is less effective” (Mother M; GNHPM, UE) |
|  |  |  | Hypothermia | “You give it to him for the fever to go down, but it almost leads to hypothermia” (Mother γ; GHPM, RE) |
|  |  | Route of administration | Oral | “She even sucks the syringe” (Father m; GHPF, UE) |
|  |  |  | Rectal | “When we were little, we were always given suppositories” (Mother R; GHPM, UE) |
|  |  | Dosing scheme | Approximate dose (without measuring it) | “I guess a more or less approximate dose [of Paracetamol]” (Mother D; GNHPM, RE) |
|  |  |  | Personalized | “I also believe it depends on the child, because there are children for whom it works and other children for whom it doesn’t work.” (Mother J; GNHPM, UE) |
|  |  |  | According to age/weight | “If it’s by age, that’s how you calculate it, or if it’s by weight you use the weight” (Mother γ; GHPM, RE) |
|  |  |  | Time interval | “Every eight hours you must give them something” (Father b; GNHPF, RE)  “Depending on how he gets by, sometimes no more than six hours” (Mother T; GHPM, UE) |
|  |  |  | Caution | “I am very cautious about medicine, I would rather give too little than give too much” (Mother C; GNHPM, RE) |
|  |  |  | Combined therapy | “If he doesn’t have many associated symptoms, first you must administrate Paracetamol and then Ibuprofen, or whatever, alternating the drugs” (Mother N; GNHPM, UE) |
|  |  | Therapeutic preferences | Metalgial | “I think that Metamizole has more of a neutral taste” (Mother O; GHPM, UE) |
|  |  |  | Dalsy | “They prefer Ibuprofen to Parcetamol” (Father r; GHPF, RE) |
|  |  |  | Paracetamol | “My daughter likes Apiretal” (Mother α; GHPM, RE) |
|  |  |  | Poor acceptance of paracetamol | “I have had many problems with paracetamol…” (Mother S; GHPM, UE) |
|  |  |  | Suppositories | “It’s a god-send, seriously, it takes away the fever fast, I don’t know if some children spit out half the medicine” (Mother F; GNHPM, RE) |
|  |  |  | Sachets | “Now that he’s older I can give it to him in the sachet and he seems to tolerate it better than Paracetamol” (Mother Y; GHPM, RE) |
|  |  |  | Sublingual | “They should invent some sublingual ones” (Mother O; GHPM, UE) |
| Non-pharmacological treatment of fever | Physical measures |  | None | “I don’t use other measures” (Father ñ; GHPF, RE) |
|  |  |  | First option | “Often, we use physical measures and the bath is a little more lukewarm than usual, and if it doesn’t work, well…” (Mother U; GHPM, RE) |
|  |  |  | Rubs | “You apply some rubs” (Mother B; GNHPM, RE) |
|  |  |  | Lukewarm baths | “A lukewarm shower” (Father o, GHPF, RE) |
|  |  |  | Cold compresses | “… and then flannels here [on the forehead]” (Mother Q; GHPM, UE) |
|  |  |  | Appropriate hydration | “Give him water with a syringe so that he doesn’t become dehydrated” (Mother F; GNHPM, RE) |
|  |  |  | Adapting clothing | “Taking off clothes so that she is more lightly dressed” (Father n; GHPF, RE) |
|  |  |  | Appropriate environment (fresh and humid) | “Even though I give him many baths I have to fan him” (Mother H; GNHPM, RE) |
|  | Natural treatment |  | Vitamin complex | “I tried it (vitamin complexes) and it worked for me” (Mother E; GNHPM, RE) |
|  |  |  | Homeopathy | “I don’t really believe in this type of treatment” (Father s; GHPF, UE)  “I found improvement with homeopathy” ((Mother H; GNHPM, RE) |
|  |  |  | Reflexology | “I took my kids to this thing where they give them a massage on the feet and it went very well… for when they were ill, for sleeping, for calming them down… during the time they went, the truth is they didn’t get ill” (Father t; GNHPF, UE) |
|  |  |  | Medicinal plants | “I give him three balls of belladonna” (Mother C; GNHPM, RE); (Mother H; GNHPM, RE) |
|  |  |  | Propolis | “Propolis, that’s what I give him” (Mother D; GNHPM, RE) |
|  | Observation |  | Co-sleeping | “If we see our little girl has had a fever all day, we bring her to bed with us” (Father s; GHPF, RE) |
|  |  |  | Night-time vigilance | “A sleepless night at home” (Father u; GNHPF, UE) |
| Influential factors in the management of fever | Family environment |  | Help from the grandmother | “I have my mother-in-law by my side and she helps me” (Father s; GHPF, UE) |
|  |  |  | Pressure from grandparents | “The grandparents… when they say. Why don’t you take him to the health clinic?... and his grandmother, poor thing, because she is so concerned and worried, they pressure us” (Mother V; GHPM, RE) |
|  |  |  | Personality of the mother | “her mother is much calmer, it seems like she controls things better...” (Father t; GNHPF, UE) |
|  |  |  | Role of the father in care | “It helps me, but I take care of most things” (Mother β; GHPM, RE) |
|  |  |  |  | “I work in the mornings and my partner works during the evenings… both her and I take turns” (Father x; GNHPF, UE) |
|  |  |  | Without the influence of other family members | “Without grandfathers or anyone close by… you have to juggle everything” (Father y; GNHPF, UE) |
|  | Age of child |  | Being able to verbally express themselves | “The age of the children also influences what they can express” (Mother J; GNHPM, UE) |
|  |  |  | Vulnerability | “They are much more vulnerable regarding everything” (Mother Y; GHPM, RE) |
|  |  |  | Different symptoms | “I noticed the symptoms were different depending on age” (Father b; GNHPF, RE) |
|  | Parents’ past experience |  | Self-learning | “We have to learn” (Father v; GNHPF, UE) |
|  |  |  | Being first-time parents | “First, we have to treat it somehow because you are a first-time mother and with the second child you already have experience” (Mother J; GNHPM, UE) |
|  |  |  | Excessive trust/ carelessness | “I realize that maybe I do too little… I don’t give it any importance, I don’t even check, I mean I don’t use the stethoscope” “I’m a bit careless” (Mother Q; GHPM, UE) |
|  |  |  | Overprotection | “Both extremes can happen, sometimes you overprotect them, and with a slight fever, 37.2 you’re already there [in the emergency room] ...” (Father p, GHPF, RE) |
|  | Lack of information |  | Treatment with antipyretics | “Does it have a sort of anesthesia [referring to Paracetamol]?” (Mother G; GNHPM, RE) |
|  |  |  | Physical measures | “Should you take off their clothes? When the temperature has gone down to 37… what do you do? Do you put her clothes back on?” “Are humidifiers good?” “In the morning, how should I dress her? With short sleeves or with a jacket?” (Father a; GNHPF, RE) |
|  | Maternal instinct |  | Innate quality | “Until now, my instinct is what has worked” (Mother A; GNHPM, RE) |
|  | Work history |  | Delegating in the father/grandparents | “My daughter spends a lot of time with the grandmother, because of my work she takes care of her and she has to take care of her when she is ill, we are dependent on the grandmother…” (Father v; GNHPF, UE) |
|  |  |  | Against school absenteeism | “The problem is when they have fever, then they can’t go to school, where do you take them because you have to go to work…” (Father y; GNHPF, UE) |
|  | Health profession |  | Particular ways of managing fever | “I think that health professional mothers hold on too much” (Mother O; GHPM, UE) |
|  |  |  | Extra responsibility in care | “My husband, the father, places all the responsibility on myself… you are the one who knows, you…” (Mother O; GHPM, UE) |
|  |  |  | Self-diagnosis | “If it goes on for longer, then I check her throat and ears” (Father r; GHPF, RE) |

Group of non-health professional mothers (GNHPM); group of non-health professional fathers (GNHPF); group of health professional mothers (GHPM); group of health professional fathers (GHPF); urban environment (UE); rural environment (RE).
